# Crystal structures of di-μ-bromido-bis{di­bromido­[η^5^-2-(di­methyl­amino)­inden­yl]zirconium(IV)} and di­bromido­bis­[η^5^-2-(di­methyl­amino)­inden­yl]zirconium(IV)

**DOI:** 10.1107/S2056989016016595

**Published:** 2016-10-21

**Authors:** Michael G. Medvedev, Ilya S. Borisov, Pavel S. Kulyabin, Vyatcheslav V. Izmer, Dmitry S. Kononovich, Dmitry V. Uborsky, Alexander Z. Voskoboynikov

**Affiliations:** a X-ray Structural Laboratory, A. N. Nesmeyanov Institute of Organoelement Compounds, Vavilova St. 28, GSP-1, Moscow 119991, V-334, Russian Federation; b Department of Chemistry, Lomonosov Moscow State University, 1/3 Leninskie Gory, GSP-1, Moscow 119991, Russian Federation

**Keywords:** crystal structure, zirconium, organozirconium compounds

## Abstract

In the title compounds, [Zr_2_Br_6_(C_11_H_12_N)_2_], (I) and [ZrBr_2_(C_11_H_12_N)_2_], (II), the positions of the η^5^-binding 2-di­methyl­amino­indenyl units are fixed by intra­molecular C—H⋯Br inter­actions involving aromatic or di­methyl­amino H atoms. The binuclear mol­ecule of (I) is located on a general position, while the mononuclear mol­ecule of (II) is situated on a twofold rotation axis. Both Zr^IV^ atoms in (I) are ligated by one cyclo­penta­dienyl (CP) ring and four Br ligands (two bridging, two terminal), while in (II) the Zr^IV^ atom is ligated by two CP rings and two terminal Br ligands. The crystal structures of both (I) and (II) comprise of strands of π–π- and N–π-bonded mol­ecules, which in turn are linked by C—H⋯Br inter­actions.

## Abstract 2

Mol­ecules of di[(μ-bromido)(η^5^-2-di­methyl­amino­inden­yl)di­bromido­zirconium(IV)], (I), and bis­(η^5^-2-di­methyl­amino­inden­yl)di­bromido­zirconium(IV), (II), are dinuclear with one CP ligand and four Br ligands for each of the Zr^IV^ atoms and mononuclear with two CP and two Br ligands for the Zr^IV^ atom, respectively.

## Chemical context   

In the course of a systematic study of the mol­ecular and crystal structures of cyclo­penta­dienyl-halogenide complexes of zirconium(IV) and hafnium(IV) bearing oxygen- and nitro­gen-containing substituents at the cyclo­penta­dienyl-type ligand(s) to understand possible intra- and inter­molecular inter­actions between the ligands resulting in specific conformational properties of the complexes as well as to explain influences of the electronic properties of the involved fragments, we have determined several new crystal structures. These results are of importance for the understanding of possible inter­molecular inter­actions in solutions of the compounds under investigation for their further use in catalysis. Here we report on synthesis and crystal structures of two Zr^IV^ complexes with substituted indenyl ligands, [Zr_2_(C_11_H_12_N)_2_Br_6_], (I) and [Zr(C_11_H_12_N)_2_Br_2_], (II). Other zirconium(IV) complexes with indenyl ligands have been reported by Chirik (2010 ▸) and Pinkas & Lamač (2015 ▸).

## Structural commentary   

Structure determination revealed that both title compounds are monomeric in the solid state, with the di­methyl­amino­indenyl anions acting as η^5^-ligands and the Zr^IV^ atoms being above the centres of cyclo­penta­dienyl (CP) rings. The 2-di­methyl­amino­indenyl units deviate from planarity, the highest deviations involving the N atoms in (I) [0.165 (3) Å for N1 in the first anion and 0.171 (3) Å for N2 in the second anion] and one C atom [0.187 (1) Å for C9] in (II). The Zr⋯centroid(CP) distances are 2.1815 (15) Å and 2.1823 (15) Å in (I) and 2.2278 (6) Å in (II). The dihedral angles between the planes of the indenyl units which belong to the same mol­ecule are 3.70 (8)° in (I) and 44.25 (5)° in (II).
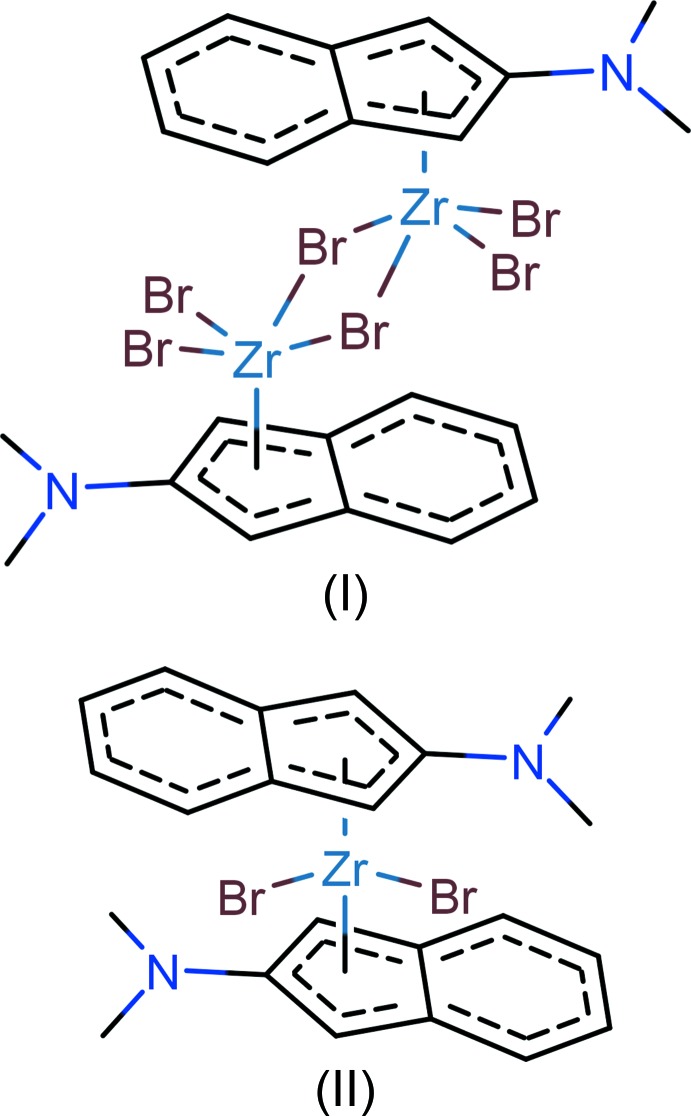


**Figure 1:**
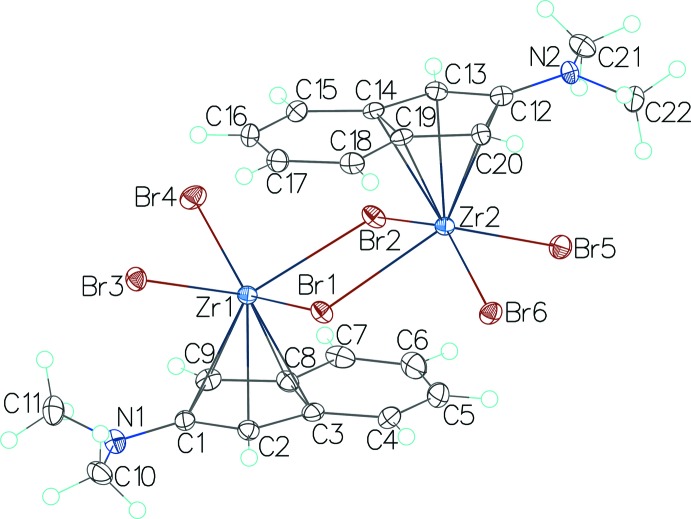
The mol­ecular structure of compound (I) with displacement ellipsoids drawn at the 50% probability level.

Compound (I) (Fig. 1 ▸) crystallizes with one binuclear complex mol­ecule in the asymmetric unit. Each of the Zr^IV^ atoms is coordinated by one CP and four Br ligands, with two Br ligands in a bridging and two in a terminal coordination mode. The Zr⋯Zr distance is 4.3359 (5) Å, a little longer than in a related complex with 2-(9*H*-carbazol-9-yl)indenyl ligands [4.3212 (7) Å; Lebedev *et al.*, 2009 ▸]. The Zr—centroid(CP) distances found in (I) are virtually identical to those of the related complex [2.1812 (15) and 2.1845 (15) Å; Lebedev *et al.*, 2009 ▸] and close to those of other similar complexes with Cl and Cp* ligands [2.176 (2) Å; Martín *et al.*, 1994 ▸] or Cl and 1-[*n*-but­yl(dimeth­yl)sil­yl]-2,3,4,5,6,7-hexa­methyl­indenyl ligands [2.1896 (8) Å; Buffet *et al.*, 2015 ▸]. In (I), the range of centroid(CP)—Zr—Br angles is 103–104° and 108–111° for bridging and terminal Br ligands, respectively. The Br—Zr—Br angles are 75.823 (13) and 76.248 (13)° for bridging Br ligands and 92.208 (16) and 90.069 (16)° for terminal Br ligands.

**Figure 2:**
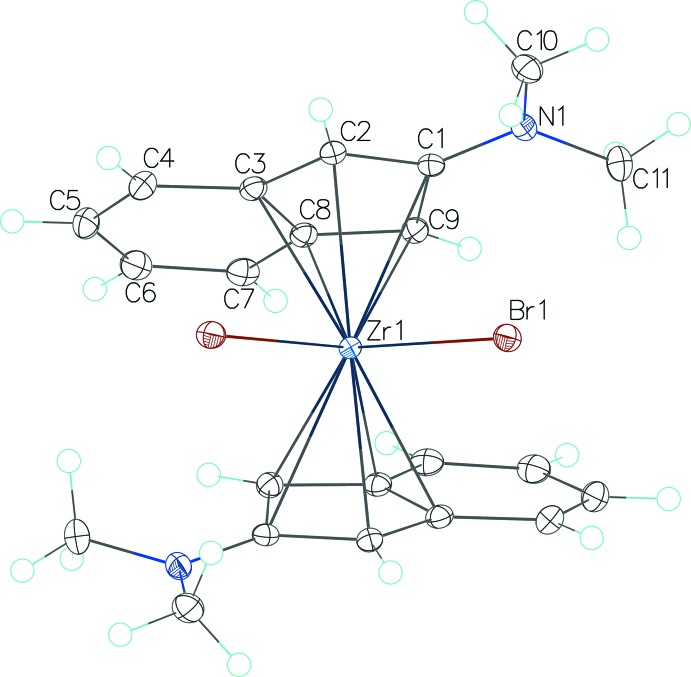
The mol­ecular structure of compound (II) with displacement ellipsoids drawn at the 50% probability level. Unlabelled atoms are generated by symmetry code −*x* + 1, *y*, −*z* + 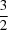.

Compound (II) (Fig. 2 ▸) crystallizes with one half of the complex mol­ecule in the asymmetric unit, the other half being completed by application of twofold rotation symmetry. Here the Zr^IV^ atom is coordinated by two symmetry-related CP ligands and two symmetry-related terminal Br ligands. The Br—Zr—Br angle is 93.390 (7)°, smaller than in related structures with Cl ligands [95.04 (8) or 94.90 (8)°; Barsties *et al.*, 1996 ▸; Luttikhedde *et al.*, 1996 ▸]. Correspondingly, the centroid(CP)—Zr—centroid(CP) angle is a little bit larger at 133.42 (3)° *versus* 133.07 (12) and 132.77 (14)° in the related structures.

The positions of the 2-di­methyl­amino­indenyl units in the two structures are fixed by intra­molecular C—H⋯Br inter­actions involving aromatic or di­methyl­amino H atoms (Tables 1 ▸ and 2 ▸).

## Supra­molecular features   

**Figure 3:**
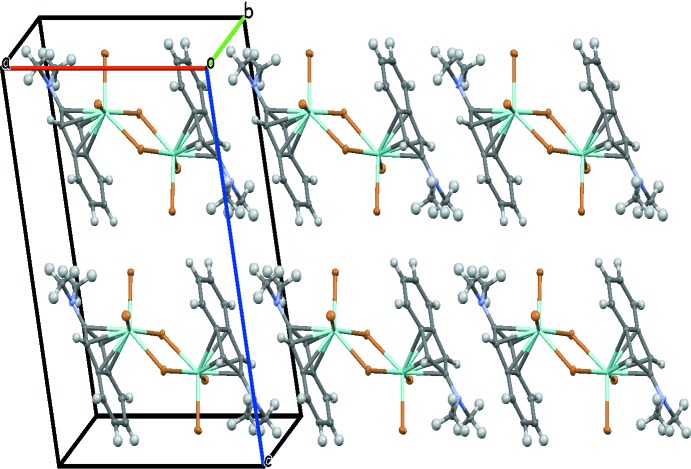
The crystal packing of compound (I) with displacement ellipsoids drawn at the 50% probability level.

The crystal structures of both (I) and (II) (Figs. 3 ▸ and 4 ▸) comprise of infinite strands (along [100] for (I) and along [001] for (II)), of π–π- and N–π-bonded mol­ecules, which in turn are linked by C—H⋯Br inter­actions. The plane-to-plane distances of the stacked di­methyl­amino­indenyl moieties are 3.656 (4) and 3.481 (3) Å for (I) and 3.6533 (10) Å for (II), with angles between the planes of 3.70 (8)° for (I) and 0° for (II). C—H⋯Br inter­actions are in the range 2.86–3.53 Å for both structures (Tables 1 ▸ and 2 ▸). The presence of the ternary amino function in the two structures plays a crucial role in the supra­molecular architecture since di­chlorido-bis­(η^5^-2-di­methyl­amino­inden­yl)zirconium(IV) (Barsties *et al.*, 1996 ▸; Luttikhedde *et al.*, 1996 ▸) also exhibits stacking inter­actions, but di­chlorido-bis­(η^5^-inden­yl)zirconium(IV) (Repo *et al.*, 1996 ▸) does not.

## Synthesis and crystallization   

Di[(μ-bromido)(η^5^-2-di­methyl­amino­inden­yl)di­bromidozirconium(IV)], (I), was obtained by reaction of Zr(NMe_2_)_4_ with one equivalent of 2-di­methyl­amino-1*H*-indene in toluene, followed by treatment of an excess of Me_3_SiBr. The crude product was recrystallized from toluene.

Bis(η^5^-2-di­methyl­amino­inden­yl)di­bromido­zirconium(IV), (II), was obtained from the reaction of (I) with one equivalent (per Zr) of 2-di­methyl­amino­indenyllithium in tetra­hydro­furan. The crude product was recrystallized from toluene.

## Refinement   

Crystal data, data collection and structure refinement details are summarized in Table 3 ▸. H atoms were fixed geometrically and refined using a riding model with *U*_iso_(H) = 1.2*U*_eq_(C) for aromatic hydrogen atoms and *U*_iso_(H) = 1.5*U*_eq_(C) for hydrogen atoms associated with methyl groups.

## Supplementary Material

Crystal structure: contains datablock(s) I, II, New_Global_Publ_Block. DOI: 10.1107/S2056989016016595/wm5329sup1.cif

CCDC references: 1510292, 1510291

Additional supporting information: crystallographic information; 3D view; checkCIF report

## Figures and Tables

**Figure 4:**
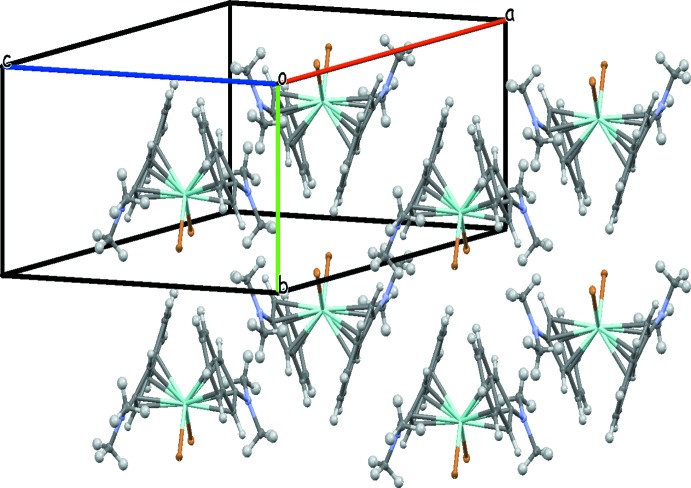
The crystal packing of compound (II) with displacement ellipsoids drawn at the 50% probability level.

**Table 1:** Hydrogen-bond geometry (Å, °) for (I)

*D*—H⋯*A*	*D*—H	H⋯*A*	*D*⋯*A*	*D*—H⋯*A*
C2—H2⋯Br4^i^	1.00	2.96	3.694 (4)	131
C4—H4⋯Br4^i^	0.95	3.35	3.944 (4)	123
C4—H4⋯Br5^ii^	0.95	3.42	4.123 (4)	132
C5—H5⋯Br5	0.95	3.53	3.998 (4)	113
C5—H5⋯Br5^ii^	0.95	3.52	4.182 (4)	129
C5—H5⋯Br6^ii^	0.95	2.93	3.840 (4)	162
C10—H10*C*⋯Br3	0.98	2.88	3.613 (4)	133
C11—H11*A*⋯Br6^iii^	0.98	2.91	3.817 (4)	154
C11—H11*B*⋯Br3	0.98	3.21	3.843 (4)	124
C11—H11*B*⋯Br5^iv^	0.98	3.44	3.919 (4)	113
C13—H13⋯Br6^v^	1.00	3.04	3.690 (4)	124
C15—H15⋯Br6^v^	0.95	3.04	3.655 (4)	124
C16—H16⋯Br3	0.95	3.27	3.763 (4)	114
C16—H16⋯Br3^vi^	0.95	3.15	3.754 (4)	123
C17—H17⋯Br3^vi^	0.95	2.97	3.665 (4)	131
C18—H18⋯Br2^vii^	0.95	3.01	3.897 (4)	155
C20—H20⋯Br4^vii^	1.00	3.17	4.115 (4)	158
C21—H21*B*⋯Br5	0.98	2.94	3.660 (4)	131
C21—H21*C*⋯Br3^viii^	0.98	3.20	3.811 (4)	122
C22—H22*A*⋯Br4^vii^	0.98	3.02	3.986 (4)	167
C22—H22*B*⋯Br4^viii^	0.98	3.36	3.974 (4)	122
C22—H22*C*⋯Br5	0.98	3.06	3.738 (4)	128

Symmetry codes: (i) 
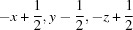
; (ii) 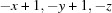; (iii) 
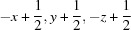
; (iv) 
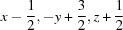
; (v) 
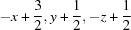
; (vi) 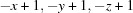; (vii) 
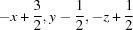
; (viii) 
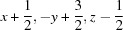
.

**Table 2:** Hydrogen-bond geometry (Å, °) for (II)

*D*—H⋯*A*	*D*—H	H⋯*A*	*D*⋯*A*	*D*—H⋯*A*
C5—H5⋯Br1^i^	0.95	2.98	3.8960 (13)	163
C6—H6⋯Br1^ii^	0.95	3.10	3.7621 (13)	128
C7—H7⋯Br1^ii^	0.95	3.09	3.7493 (12)	128
C10—H10*A*⋯Br1^iii^	0.98	3.11	3.9381 (13)	143
C10—H10*C*⋯Br1	0.98	2.86	3.5178 (13)	125
C11—H11*A*⋯Br1^iv^	0.98	3.41	3.9170 (13)	115
C11—H11*B*⋯Br1	0.98	3.04	3.6325 (13)	120

Symmetry codes: (i) 
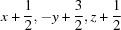
; (ii) 
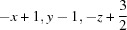
; (iii) 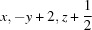; (iv) 
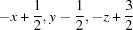
.

**Table 3:** Experimental details

	(I)	(II)
Crystal data		
Chemical formula	[Zr_2_Br_6_(C_11_H_12_N)_2_]	[ZrBr_2_(C_11_H_12_N)_2_]
*M*_r_	978.33	567.47
Crystal system, space group	Monoclinic, *P*2_1_/*n*	Monoclinic, *C*2/*c*
Temperature (K)	100	100
*a*, *b*, *c* (Å)	11.3275 (6), 13.9365 (7), 17.6082 (9)	18.4476 (5), 8.3497 (2), 14.3737 (4)
β (°)	99.028 (1)	111.854 (1)
*V* (Å^3^)	2745.3 (2)	2054.90 (9)
*Z*	4	4
Radiation type	Mo *K*α	Mo *K*α
μ (mm^−1^)	9.51	4.43
Crystal size (mm)	0.34 × 0.14 × 0.04	0.26 × 0.24 × 0.15

Data collection		
Diffractometer	Bruker APEXII CCD	Bruker APEXII CCD
Absorption correction	Multi-scan (*SADABS*; Bruker, 2008 ▸)	Multi-scan (*SADABS*; Bruker, 2008 ▸)
*T*_min_, *T*_max_	0.052, 0.165	0.552, 0.747
No. of measured, independent and observed [*I* > 2σ(*I*)] reflections	35046, 7921, 6465	13152, 3003, 2876
*R*_int_	0.050	0.017
(sin θ/λ)_max_ (Å^−1^)	0.703	0.703

Refinement		
*R*[*F* ^2^ > 2σ(*F* ^2^)], *wR*(*F* ^2^), *S*	0.033, 0.075, 1.04	0.015, 0.039, 1.07
No. of reflections	7921	3003
No. of parameters	293	125
H-atom treatment	H-atom parameters constrained	H-atom parameters constrained
Δρ_max_, Δρ_min_ (e Å^−3^)	1.23, −0.80	0.45, −0.42

Computer programs: *APEX2* (Bruker, 2012 ▸), *SAINT* (Bruker, 2009 ▸), *SHELXT* (Sheldrick, 2015*a* ▸), *SHELXL2014* (Sheldrick, 2015*b* ▸) and *OLEX2* (Dolomanov *et al.*, 2009 ▸).

## supplementary crystallographic information

### Crystal data

**Table:**

[ZrBr_2_(C_11_H_12_N)_2_]	*F*(000) = 1120
*M**_r_* = 567.47	*D*_x_ = 1.834 Mg m^−^^3^
Monoclinic, *C*2/*c*	Mo *K*α radiation, λ = 0.71073 Å
*a* = 18.4476 (5) Å	Cell parameters from 9893 reflections
*b* = 8.3497 (2) Å	θ = 2.7–35.7°
*c* = 14.3737 (4) Å	µ = 4.43 mm^−^^1^
β = 111.854 (1)°	*T* = 100 K
*V* = 2054.90 (9) Å^3^	Prism, yellow
*Z* = 4	0.26 × 0.24 × 0.15 mm

### Data collection

**Table:**

Bruker APEXII CCD diffractometer	2876 reflections with *I* > 2σ(*I*)
Graphite monochromator	*R*_int_ = 0.017
φ and ω scans	θ_max_ = 30.0°, θ_min_ = 2.4°
Absorption correction: multi-scan (*SADABS*; Bruker, 2008)	*h* = −25→25
*T*_min_ = 0.552, *T*_max_ = 0.747	*k* = −11→11
13152 measured reflections	*l* = −20→20
3003 independent reflections	

### Refinement

**Table:**

Refinement on *F*^2^	Primary atom site location: dual
Least-squares matrix: full	Hydrogen site location: inferred from neighbouring sites
*R*[*F*^2^ > 2σ(*F*^2^)] = 0.015	H-atom parameters constrained
*wR*(*F*^2^) = 0.039	*w* = 1/[σ^2^(*F*_o_^2^) + (0.0186*P*)^2^ + 2.1368*P*] where *P* = (*F*_o_^2^ + 2*F*_c_^2^)/3
*S* = 1.07	(Δ/σ)_max_ = 0.001
3003 reflections	Δρ_max_ = 0.45 e Å^−^^3^
125 parameters	Δρ_min_ = −0.42 e Å^−^^3^
0 restraints	

### Special details

**Table:**

Geometry. All esds (except the esd in the dihedral angle between two l.s. planes) are estimated using the full covariance matrix. The cell esds are taken into account individually in the estimation of esds in distances, angles and torsion angles; correlations between esds in cell parameters are only used when they are defined by crystal symmetry. An approximate (isotropic) treatment of cell esds is used for estimating esds involving l.s. planes.

### Fractional atomic coordinates and isotropic or equivalent isotropic displacement parameters (Å^2^)

**Table:**

	*x*	*y*	*z*	*U*_iso_*/*U*_eq_	
Zr1	0.5000	0.74647 (2)	0.7500	0.00832 (4)	
Br1	0.38964 (2)	0.96156 (2)	0.68020 (2)	0.01412 (4)	
N1	0.35083 (6)	0.76823 (12)	0.85552 (8)	0.01326 (19)	
C1	0.42107 (7)	0.70340 (14)	0.86778 (8)	0.0114 (2)	
C2	0.49565 (7)	0.77305 (14)	0.92202 (9)	0.0116 (2)	
H2	0.5045	0.8746	0.9616	0.014*	
C3	0.55415 (7)	0.65358 (14)	0.93154 (8)	0.0120 (2)	
C4	0.63626 (7)	0.65095 (16)	0.98526 (9)	0.0156 (2)	
H4	0.6625	0.7421	1.0221	0.019*	
C5	0.67705 (7)	0.51419 (17)	0.98303 (10)	0.0175 (2)	
H5	0.7319	0.5112	1.0190	0.021*	
C6	0.63885 (7)	0.37755 (16)	0.92804 (9)	0.0167 (2)	
H6	0.6684	0.2839	0.9293	0.020*	
C7	0.55973 (7)	0.37769 (14)	0.87287 (9)	0.0148 (2)	
H7	0.5348	0.2857	0.8358	0.018*	
C8	0.51605 (7)	0.51796 (14)	0.87243 (9)	0.0116 (2)	
C9	0.43396 (7)	0.55692 (14)	0.82397 (9)	0.0119 (2)	
H9	0.3922	0.4798	0.7851	0.014*	
C10	0.34843 (7)	0.92403 (15)	0.89929 (10)	0.0166 (2)	
H10A	0.3813	0.9222	0.9708	0.025*	
H10B	0.2945	0.9492	0.8911	0.025*	
H10C	0.3678	1.0058	0.8655	0.025*	
C11	0.28038 (7)	0.69950 (17)	0.78212 (10)	0.0184 (2)	
H11A	0.2784	0.5848	0.7953	0.028*	
H11B	0.2807	0.7148	0.7147	0.028*	
H11C	0.2345	0.7526	0.7868	0.028*	

### Atomic displacement parameters (Å^2^)

**Table:**

	*U*^11^	*U*^22^	*U*^33^	*U*^12^	*U*^13^	*U*^23^
Zr1	0.00882 (7)	0.00802 (7)	0.00796 (7)	0.000	0.00294 (5)	0.000
Br1	0.01459 (6)	0.01476 (6)	0.01381 (6)	0.00574 (4)	0.00618 (4)	0.00414 (4)
N1	0.0113 (4)	0.0155 (5)	0.0137 (4)	−0.0001 (4)	0.0054 (4)	−0.0013 (4)
C1	0.0125 (5)	0.0128 (5)	0.0098 (5)	−0.0001 (4)	0.0054 (4)	0.0011 (4)
C2	0.0119 (5)	0.0130 (5)	0.0097 (5)	−0.0002 (4)	0.0041 (4)	−0.0013 (4)
C3	0.0134 (5)	0.0132 (5)	0.0095 (5)	0.0007 (4)	0.0043 (4)	0.0015 (4)
C4	0.0139 (5)	0.0193 (6)	0.0119 (5)	0.0001 (4)	0.0028 (4)	0.0008 (4)
C5	0.0132 (5)	0.0230 (6)	0.0150 (5)	0.0037 (5)	0.0037 (4)	0.0046 (5)
C6	0.0190 (6)	0.0163 (6)	0.0163 (5)	0.0067 (4)	0.0082 (5)	0.0058 (4)
C7	0.0192 (5)	0.0120 (5)	0.0142 (5)	0.0025 (4)	0.0076 (4)	0.0029 (4)
C8	0.0133 (5)	0.0115 (5)	0.0103 (5)	0.0007 (4)	0.0048 (4)	0.0022 (4)
C9	0.0125 (5)	0.0115 (5)	0.0115 (5)	−0.0011 (4)	0.0045 (4)	0.0003 (4)
C10	0.0169 (5)	0.0177 (6)	0.0168 (5)	0.0035 (4)	0.0081 (4)	−0.0015 (4)
C11	0.0112 (5)	0.0225 (6)	0.0200 (6)	−0.0021 (5)	0.0042 (4)	−0.0007 (5)

### Geometric parameters (Å, º)

**Table:**

Zr1—Br1	2.6183 (2)	C3—C4	1.4215 (16)
Zr1—Br1^i^	2.6183 (2)	C3—C8	1.4330 (16)
Zr1—C1	2.6365 (11)	C4—H4	0.9500
Zr1—C1^i^	2.6365 (11)	C4—C5	1.3745 (18)
Zr1—C2	2.5134 (11)	C5—H5	0.9500
Zr1—C2^i^	2.5134 (11)	C5—C6	1.4171 (19)
Zr1—C3^i^	2.5432 (11)	C6—H6	0.9500
Zr1—C3	2.5432 (11)	C6—C7	1.3775 (17)
Zr1—C8	2.5371 (11)	C7—H7	0.9500
Zr1—C8^i^	2.5371 (11)	C7—C8	1.4203 (16)
Zr1—C9	2.4666 (12)	C8—C9	1.4488 (16)
Zr1—C9^i^	2.4665 (12)	C9—H9	1.0000
N1—C1	1.3538 (15)	C10—H10A	0.9800
N1—C10	1.4529 (16)	C10—H10B	0.9800
N1—C11	1.4528 (16)	C10—H10C	0.9800
C1—C2	1.4284 (16)	C11—H11A	0.9800
C1—C9	1.4355 (16)	C11—H11B	0.9800
C2—H2	1.0000	C11—H11C	0.9800
C2—C3	1.4382 (16)		
			
Br1^i^—Zr1—Br1	93.390 (7)	C9—Zr1—C8^i^	82.39 (4)
Br1^i^—Zr1—C1	112.58 (3)	C9^i^—Zr1—C8	82.39 (4)
Br1^i^—Zr1—C1^i^	78.64 (3)	C9^i^—Zr1—C8^i^	33.62 (4)
Br1—Zr1—C1	78.64 (3)	C9^i^—Zr1—C9	100.17 (6)
Br1—Zr1—C1^i^	112.58 (3)	C1—N1—C10	118.92 (10)
C1—Zr1—C1^i^	164.32 (5)	C1—N1—C11	119.38 (10)
C2^i^—Zr1—Br1^i^	90.65 (3)	C11—N1—C10	120.33 (10)
C2—Zr1—Br1^i^	82.39 (3)	N1—C1—Zr1	126.33 (8)
C2—Zr1—Br1	90.65 (3)	N1—C1—C2	126.08 (11)
C2^i^—Zr1—Br1	82.39 (3)	N1—C1—C9	126.19 (11)
C2—Zr1—C1	32.09 (4)	C2—C1—Zr1	69.20 (6)
C2^i^—Zr1—C1^i^	32.09 (4)	C2—C1—C9	107.64 (10)
C2—Zr1—C1^i^	150.61 (4)	C9—C1—Zr1	67.25 (6)
C2^i^—Zr1—C1	150.61 (4)	Zr1—C2—H2	125.2
C2—Zr1—C2^i^	169.87 (5)	C1—C2—Zr1	78.70 (6)
C2—Zr1—C3^i^	153.14 (4)	C1—C2—H2	125.2
C2^i^—Zr1—C3	153.14 (4)	C1—C2—C3	107.80 (10)
C2—Zr1—C3	33.04 (4)	C3—C2—Zr1	74.62 (6)
C2^i^—Zr1—C3^i^	33.04 (4)	C3—C2—H2	125.2
C2—Zr1—C8^i^	135.28 (4)	C2—C3—Zr1	72.34 (6)
C2—Zr1—C8	54.71 (4)	C4—C3—Zr1	119.62 (8)
C2^i^—Zr1—C8	135.28 (4)	C4—C3—C2	132.26 (11)
C2^i^—Zr1—C8^i^	54.71 (4)	C4—C3—C8	119.88 (11)
C3—Zr1—Br1^i^	82.16 (3)	C8—C3—Zr1	73.38 (6)
C3^i^—Zr1—Br1	82.16 (3)	C8—C3—C2	107.86 (10)
C3—Zr1—Br1	123.69 (3)	C3—C4—H4	120.6
C3^i^—Zr1—Br1^i^	123.69 (3)	C5—C4—C3	118.83 (12)
C3—Zr1—C1	53.09 (4)	C5—C4—H4	120.6
C3^i^—Zr1—C1^i^	53.09 (4)	C4—C5—H5	119.4
C3^i^—Zr1—C1	121.15 (4)	C4—C5—C6	121.26 (11)
C3—Zr1—C1^i^	121.16 (4)	C6—C5—H5	119.4
C3—Zr1—C3^i^	144.49 (5)	C5—C6—H6	119.3
C8^i^—Zr1—Br1	112.13 (3)	C7—C6—C5	121.39 (11)
C8^i^—Zr1—Br1^i^	130.91 (3)	C7—C6—H6	119.3
C8—Zr1—Br1^i^	112.13 (3)	C6—C7—H7	120.6
C8—Zr1—Br1	130.91 (3)	C6—C7—C8	118.75 (11)
C8^i^—Zr1—C1^i^	53.26 (4)	C8—C7—H7	120.6
C8—Zr1—C1	53.26 (4)	C3—C8—Zr1	73.85 (6)
C8—Zr1—C1^i^	113.14 (4)	C3—C8—C9	107.57 (10)
C8^i^—Zr1—C1	113.14 (4)	C7—C8—Zr1	122.88 (8)
C8^i^—Zr1—C3	112.47 (4)	C7—C8—C3	119.78 (11)
C8—Zr1—C3^i^	112.47 (4)	C7—C8—C9	132.61 (11)
C8—Zr1—C3	32.77 (4)	C9—C8—Zr1	70.52 (6)
C8^i^—Zr1—C3^i^	32.77 (4)	Zr1—C9—H9	125.1
C8^i^—Zr1—C8	82.46 (5)	C1—C9—Zr1	80.30 (7)
C9^i^—Zr1—Br1^i^	99.61 (3)	C1—C9—C8	107.14 (10)
C9—Zr1—Br1	99.61 (3)	C1—C9—H9	125.1
C9—Zr1—Br1^i^	135.50 (3)	C8—C9—Zr1	75.86 (7)
C9^i^—Zr1—Br1	135.50 (3)	C8—C9—H9	125.1
C9—Zr1—C1^i^	131.97 (4)	N1—C10—H10A	109.5
C9—Zr1—C1	32.46 (4)	N1—C10—H10B	109.5
C9^i^—Zr1—C1	131.97 (4)	N1—C10—H10C	109.5
C9^i^—Zr1—C1^i^	32.46 (4)	H10A—C10—H10B	109.5
C9—Zr1—C2^i^	133.04 (4)	H10A—C10—H10C	109.5
C9^i^—Zr1—C2	133.04 (4)	H10B—C10—H10C	109.5
C9—Zr1—C2	55.31 (4)	N1—C11—H11A	109.5
C9^i^—Zr1—C2^i^	55.31 (4)	N1—C11—H11B	109.5
C9—Zr1—C3	55.28 (4)	N1—C11—H11C	109.5
C9^i^—Zr1—C3	100.26 (4)	H11A—C11—H11B	109.5
C9^i^—Zr1—C3^i^	55.28 (4)	H11A—C11—H11C	109.5
C9—Zr1—C3^i^	100.26 (4)	H11B—C11—H11C	109.5
C9—Zr1—C8	33.62 (4)		
			
Zr1—C1—C2—C3	−69.62 (8)	C3—C8—C9—Zr1	65.07 (8)
Zr1—C1—C9—C8	71.81 (8)	C3—C8—C9—C1	−9.88 (13)
Zr1—C2—C3—C4	114.30 (13)	C4—C3—C8—Zr1	−115.04 (10)
Zr1—C2—C3—C8	−65.30 (8)	C4—C3—C8—C7	3.99 (17)
Zr1—C3—C4—C5	−90.04 (13)	C4—C3—C8—C9	−177.92 (10)
Zr1—C3—C8—C7	119.04 (11)	C4—C5—C6—C7	1.52 (19)
Zr1—C3—C8—C9	−62.88 (8)	C5—C6—C7—C8	−0.58 (18)
Zr1—C8—C9—C1	−74.95 (8)	C6—C7—C8—Zr1	87.19 (13)
N1—C1—C2—Zr1	−120.49 (11)	C6—C7—C8—C3	−2.14 (17)
N1—C1—C2—C3	169.89 (11)	C6—C7—C8—C9	−179.66 (12)
N1—C1—C9—Zr1	119.29 (11)	C7—C8—C9—Zr1	−117.19 (13)
N1—C1—C9—C8	−168.89 (11)	C7—C8—C9—C1	167.86 (12)
C1—C2—C3—Zr1	72.44 (8)	C8—C3—C4—C5	−3.07 (17)
C1—C2—C3—C4	−173.26 (12)	C9—C1—C2—Zr1	56.29 (8)
C1—C2—C3—C8	7.14 (13)	C9—C1—C2—C3	−13.33 (13)
C2—C1—C9—Zr1	−57.49 (8)	C10—N1—C1—Zr1	−88.58 (12)
C2—C1—C9—C8	14.32 (12)	C10—N1—C1—C2	0.89 (17)
C2—C3—C4—C5	177.38 (12)	C10—N1—C1—C9	−175.32 (11)
C2—C3—C8—Zr1	64.61 (8)	C11—N1—C1—Zr1	78.11 (13)
C2—C3—C8—C7	−176.35 (10)	C11—N1—C1—C2	167.58 (11)
C2—C3—C8—C9	1.73 (13)	C11—N1—C1—C9	−8.63 (18)
C3—C4—C5—C6	0.36 (18)		

Symmetry code: (i) −*x*+1, *y*, −*z*+3/2.

### Hydrogen-bond geometry (Å, º)

**Table:**

*D*—H···*A*	*D*—H	H···*A*	*D*···*A*	*D*—H···*A*
C5—H5···Br1^ii^	0.95	2.98	3.8960 (13)	163
C6—H6···Br1^iii^	0.95	3.10	3.7621 (13)	128
C7—H7···Br1^iii^	0.95	3.09	3.7493 (12)	128
C10—H10*A*···Br1^iv^	0.98	3.11	3.9381 (13)	143
C10—H10*C*···Br1	0.98	2.86	3.5178 (13)	125
C11—H11*A*···Br1^v^	0.98	3.41	3.9170 (13)	115
C11—H11*B*···Br1	0.98	3.04	3.6325 (13)	120

Symmetry codes: (ii) *x*+1/2, −*y*+3/2, *z*+1/2; (iii) −*x*+1, *y*−1, −*z*+3/2; (iv) *x*, −*y*+2, *z*+1/2; (v) −*x*+1/2, *y*−1/2, −*z*+3/2.
